# A low cost model for teaching tendon repair

**DOI:** 10.1308/003588412X13373405385214o

**Published:** 2012-07

**Authors:** MD Wijeratna, T Halsey, P Johnston

**Affiliations:** Cambridge University Hospitals NHS Foundation Trust,UK

## BACKGROUND

A recent technical note suggested that a drinking straw is a suitable model for the teaching of tendon repair and offered advantages over silicone rods.^1^ We have developed an alternative model based on the drinking straw using silicone sealant.

## TECHNIQUE

**Figure 1:**
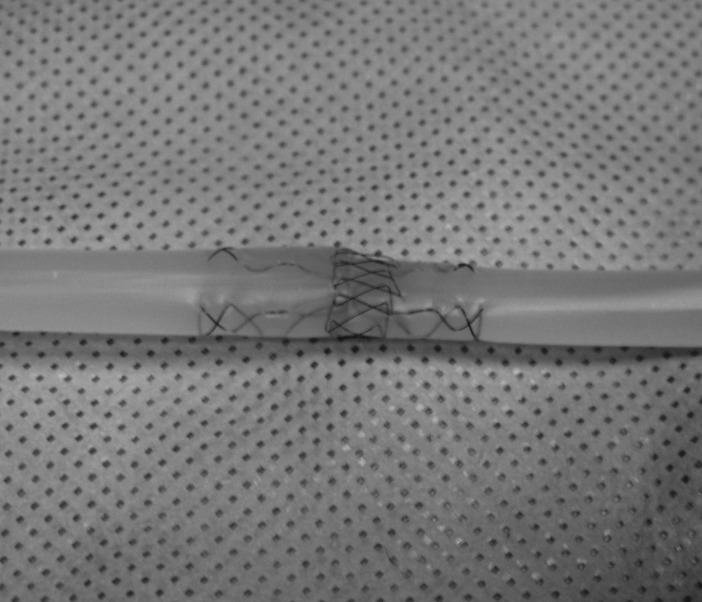
Silicone model for the teaching of tendon repair

Easily available commercial silicone sealant is used to create the model (No Nonsense® Sanitary Silicone Clear; Screwfix, Yeovil, UK). A standard drinking straw is filled from one end with silicone sealant. Under pressure, the sealant will flow to the opposite end of the straw. The sealant is then left to cure for two weeks. The model should be left in a well ventilated area as acetic acid is produced during the curing process. Once set, the drinking straw can be cut away from the model (Fig 1).

## DISCUSSION

We have found this model provides better suture handling feedback than the drinking straw model described previously.^1^ The silicone model maintains the position of inserted suture material and the accuracy of insertion can be assessed by a trainer as the model is transparent. The ‘feel’ of the model imitates that of a human tendon more realistically and marks made by the injudicious use of forceps during tendon handling are also seen easily. Different sizes of model can be made to replicate biological structures of differing diameters by using straws of varying size. A 310ml cartridge of sealant can be purchased for £1.76. This will produce over 30 lengths of the model. This is in comparison with commercially available medical grade silicone rods that cost over £150 for a 5cm length.
